# Supplementary material for: A 6-CpG validated methylation risk score model for metabolic syndrome: The HyperGEN and GOLDN studies
Source: PLoS One. 2021 Nov 15;16(11):e0259836. doi: 10.1371/journal.pone.0259836 (PMC8592434; doi:10.1371/journal.pone.0259836)
Supplement: Attachment — Submitted filename: Reviews for MetS paper_clean.docx [file pone.0259836.s001.docx]

We thank the reviewers for their thorough review of our paper and the astute feedback provided. We have addressed the reviewers’ comments as follows:

Responses to Reviewer Comments

Reviewer #1: 
Major comments

1. “…lack of detail regarding the selection process for the six CpGs.” We thank the reviewer for highlighting this. **We have added additional details about our CpG selection process to the Methods section, specifically the subsection titled ‘CgG candidate selection’. (lines 170-188)**

2. “…discussion needs to be rewritten and expanded upon in more detail.” **We have added additional context to the discussion section accordingly. (lines 292-315 and 341-345)**

3. “…running a set of secondary analyses. Specifically, similar models as for Table 4, but instead of MetS as the dichotomous outcome use each of the individual components of MetS and model them separately.” **We performed additional analyses and have subsequently updated Table 2 and Table 4. In our prior work, we have shown the robustness of CpGs included in our score with each of the MetS components, as well as with MetS. We have included references to those studies in Table 1, including directions of effect and relevant references.**

Minor comments:
1. “Consider adding the MRS risk score to Table 1, as some descriptive analysis will be a good way to assess its performance.” **We have included the MRS risk score to table 2, and have included all references of published methylation risk scores (MRS) to Table 1.**

2. “The methods section for HyperGEN should specify the source tissue for the DNA. It’s pretty clear elsewhere that this is blood, but it still should be specified in the methods.” **We have updated the methods section to make this detail clearer.** **(line 158)**

3. “On page 8 the authors refer to Table 1 when talking about the consistency between their results and the prior literature, in terms of direction of association. However, this information is not presented in Table 1- only the citations are. The authors should either a) add this information to Table 1, or b) cite specific studies and not Table 1 when making this point in the discussion. **We have added further details about each of the components and relevant literature to the text (lines 92-96), added results from analyses performed in each of the components, as well as results from our sex-stratified analyses. (Table 4)**

4. “Why was model fit in Table 5 compared in GOLDN but not HyperGEN?” **We have removed this information to avoid confusion about model fits, and have added results for additional analyses performed in tables 2 and 4.**

5. “The authors may want to consider de-emphasizing the model fit results in their concluding paragraph. I’m not sure a ~70-point difference in AIC/BIC is worth making it a primary conclusion, even if does represent an improvement in fit.” **We have made changes to the conclusion paragraph accordingly.** **(line 356)**

Reviewer #2: “…there are no relevant concerns to be addressed, I have only minor comments”:

1. “The main objective should be stated more clearly in both abstract and introduction.” We thank the reviewer for this recommendation. **We have updated the text in the introduction and abstract to make this point clearer. (lines 35-37 and lines 74-75)**

2. “I suggest to include a paragraph in the introduction section regarding to the association between epigenetic mechanisms, mainly methylation marks, on the obesity and metabolic syndrome to highlight the rationale of this study more in depth. Some of these studies could be on help (PMID: 28211912, PMID: 27477082, PMID: 30887406).” We thank the reviewer for the suggested references. **We have integrated them into both the introduction section and Table 1 of our paper. (line 82 and Table 1)**

Reviewer #3: It is a very well written article and authors should be commended for this. Few comments
1. “Since MetS parameters are known to show differential patterns in sex, and owing to the fact both HYPER AND GENO studies have higher female participants-i would like authors to discuss more about how sexual dimorphism could have impacted the MRS score.” **We performed these additional analyses and have added the results to the paper.** **(lines 218-219, 255-260, and 305-315)**

2. “The authors should also comment why only one cell type was used in the validation but not all or the vice versa, because one would assume that the gene expression would alter between different cell types.” **We agree with the reviewer that additional cell types would have been important to study, however, for this study we only had one cell type available in both HyperGEN and GOLDN.** **We have expanded on this point in the discussion section of the paper. (lines 341-345)**

3. “The authors tend to ignore referencing the facts throughout the study, I would like the authors to please go back to the introduction and discussion and use appropriate references.” **We regret that we did not include this information previously, and have added this literature and relevant references throughout, including Table 1 of the paper.**
